# Announcement

**Published:** 2015-05-15

## American Stroke Month and National High Blood Pressure Education Month — May 2015

May 2015 is American Stroke Month and National High Blood Pressure Education Month. American Stroke Month raises awareness of the signs and symptoms of stroke and encourages persons to act F.A.S.T.* (Face drooping, Arm weakness, Speech difficulty, Time to call 9–1–1) if someone is having a stroke. Stroke is the fifth leading cause of death in the United States and a leading cause of severe disability (1,2). In the United States, on average, one person dies from stroke every 4 minutes (2).

Stroke is largely preventable. You may be able to prevent stroke or reduce your risk through healthy lifestyle changes. High blood pressure is one of the major risk factors for stroke. Others include high cholesterol, smoking, diabetes, heart disease, obesity, physical inactivity, and unhealthy diet.†

National High Blood Pressure Education Month focuses on saving lives by increasing awareness and educating the public about cardiovascular disease risks and how to prevent them. High blood pressure, also known as hypertension, is considered the “silent killer” because it can damage the heart, brain, and kidneys without any symptoms (2). In the United States, nearly one in three adults has hypertension, and only about half have their condition under control (3). Hypertension is the leading risk factor for stroke and heart attacks. Each day in the United States, more than 1,000 deaths are associated with hypertension (2).

To control hypertension and reduce their risk for stroke, patients can take medications as directed, monitor their blood pressure, and eat a lower-sodium diet and more fruits and vegetables. Health care providers can use electronic health records, blood pressure measurement, and a team-based care approach to help improve their patients’ hypertension control. Patients and providers can find more information at http://millionhearts.hhs.gov/abouthds/prevention.html.

CDC focuses on promoting cardiovascular health, improving quality of care for all, and eliminating disparities associated with heart disease and stroke. Additional information is available at http://www.cdc.gov/bloodpressure and http://www.cdc.gov/stroke.

## Footnotes

* More information is available at http://www.strokeassociation.org/STROKEORG/AboutStroke/AmericanStrokeMonth/American-Stroke-Month_UCM_459942_SubHomePage.jsp.

† More information is available at http://www.cdc.gov/stroke/risk_factors.htm.
